# Co-designing and piloting adolescent-adapted social rhythm therapy for young people with unipolar and bipolar depression: a mixed-methods feasibility case series

**DOI:** 10.3389/frcha.2026.1909726

**Published:** 2026-08-28

**Authors:** Gonzalo Salazar de Pablo, Claudia Aymerich, Zahra Kamran, Cecilia Sanjuan-Ortiz, Nuria Laherran-Cantera, Shantal Zeb, Eleonora Armyra, Allan H. Young, Aditya Sharma, Cornelius Ani, Manasi Kumar, Holly A. Swartz

**Affiliations:** 1 Department of Child and Adolescent Psychiatry, Institute of Psychiatry, Psychology & Neuroscience, King’s College London, London, United Kingdom; 2 Child and Adolescent Mental Health Services, South London and Maudsley NHS Foundation Trust, London, United Kingdom; 3 Department of Child and Adolescent Psychiatry, Institute of Psychiatry and Mental Health. Hospital General La Paz School of Medicine, Universidad Complutense, IiSGM, CIBERSAM, Madrid, Spain; 4 Psychiatry Department, Biocruces Bizkaia Health Research Institute, OSI Bilbao- Basurto. Centro de Investigación en Red de Salud Mental, (CIBERSAM), Instituto de Salud Carlos III, Barakaldo, Bizkaia, Spain; 5 Department of Child and Adolescent Psychiatry, La Fe University and Polytechnic Hospital, València, Spain; 6 Department of Medicine, University of Valencia, Valencia, Spain; 7 Department of Psychiatry, Universidad de Alcalá, Madrid, Spain; 8 Unidad de Gestión Clínica de Salud Mental, Instituto de Investigación e Innovación Biomédica de Cádiz (INiBICA), Cadiz, Spain; 9 National Institute for Health Research (NIHR) Biomedical Research Centre, South London and Maudsley NHS Foundation Trust, London, United Kingdom; 10 Centre for Affective Disorders (CfAD), Institute of Psychiatry, Psychology and Neuroscience (IoPPN), King’s College London, London, United Kingdom; 11 South London and Maudsley NHS Foundation Trust, London, United Kingdom; 12 Academic Psychiatry, Translational and Clinical Research Institute, Newcastle University, Newcastle upon Tyne, United Kingdom; 13 Specialist Adolescent Mood Disorders Service (SAMS), Cumbria, Northumberland, Tyne and Wear NHS Foundation Trust, Newcastle upon Tyne, United Kingdom; 14 Division of Psychiatry, Imperial College London, London, United Kingdom; 15 Surrey and Borders Partnership NHS Foundation Trust, Leatherhead, United Kingdom; 16 University of Medical Sciences, Ondo, Nigeria; 17 Institute for Excellence in Health Equity, Department of Population Health, New York University Grossman School of Medicine, New York, NY, United States; 18 Department of Psychiatry, University of Nairobi, Nairobi, Kenya; 19 Department of Psychiatry, University of Pittsburgh School of Medicine, Pittsburgh, PA, United States

**Keywords:** adolescents, bipolar disorder, co-designing, depression, social rhythm

## Abstract

**Objective:**

Depressive and bipolar disorders commonly emerge during adolescence and are associated with disturbances in sleep, daily routines and social rhythms, as well as functional impairment. Social Rhythm Therapy (SRT) targets these processes. However, adult-derived rhythm protocols do not adequately reflect adolescents’ school and family schedules, developmental autonomy, social and digital routines, or preferences regarding monitoring. Direct youth co-design is therefore needed to improve developmental fit, engagement and feasibility. We addressed this gap by co-designing the Social Therapy for Adolescent Rhythms in Depression (STAR-D), an adolescent-adapted SRT intervention, and evaluating its feasibility and acceptability within routine Child and Adolescent Mental Health Services (CAMHS).

**Methods:**

A two-phase mixed-methods feasibility design was used. Phase 1 involved co-production and intervention adaptation using questionnaire feedback from 10 adolescents, an in-depth interview with one Young People's Advisory Group representative, and feedback from six clinicians. TIDieR principles guided intervention description and adaptation. Phase 2 piloted STAR-D in adolescents receiving care in Lambeth CAMHS, London, UK. Feasibility was assessed through referral, uptake, retention and completion. Acceptability was assessed through qualitative feedback. Exploratory clinical outcomes were described using measures of functioning, depressive symptoms and manic symptoms.

**Results:**

Phase 1 feedback identified priorities for intervention adaptation, including accessible language, explicit links between routines and mood, personalised goals and flexible delivery. These findings informed an eight-session STAR-D protocol. In Phase 2, seven young people were referred, six accepted and started STAR-D, and five completed all eight sessions. Participants described the intervention as supportive, collaborative and accessible. However, they suggested simplifying daily monitoring to reduce burden. Quantitative completion and missingness data for monitoring forms were not prospectively collected. Exploratory clinical trajectories were variable.

**Conclusion:**

STAR-D was feasible to deliver and acceptable to most participants in this small, uncontrolled, single-service pilot. The findings support further optimisation, particularly of routine monitoring, followed by a larger multicentre controlled mechanistic trial. Future work should test whether changes in sleep-wake regularity, daily routines and social rhythm stability mediate changes in mood and functioning, and should evaluate unipolar and bipolar presentations with sufficient diagnostic stratification.

## Introduction

Depressive disorders in adolescence are common, disabling and often recurrent (1, 2). They affect education, family life, peer relationships, sleep, daily functioning and long-term health (2, 3). Young people with depressive disorders frequently experience impaired concentration, low energy, school absence, reduced social participation, family stress and increased risk of self-harm or relapse (1, 2). Bipolar disorder (BD) is a chronic, recurrent and debilitating illness that is characterised by fluctuations in mood states and energy (4). BD is associated with significant impairments in cognitive, social, and everyday functioning (5) and is ranked as the illness with the second greatest effect on days out of role functioning (6), contributing to 9.9 million disability adjusted life years (DALYs) (7).

Depression is associated with psychological and contextual risk factors in young people (8), including belonging to an ethnic minority group (9), parental factors (10), early life adversity (11), bullying (12) and excessive use of social media (13). BD is also associated with a wide range of risk factors, including family history of bipolar disorder (14, 15), obstetric complications (16) and adverse life events (17). BD most often emerges in young people, often during adolescence (18). Early-onset depressive disorders and BD are associated with a more severe illness trajectory, higher rates of comorbid psychiatric conditions, and persistent functional impairments than late-onset depression, underscoring the importance of early identification and developmentally tailored interventions (18–20).

For young people with depression, psychological interventions are an essential part of long-term management to support symptom stabilisation, and thus first line treatments according to National Institute for Health and Care Excellence (NICE) Guidelines (21, 22). Psychological interventions can be beneficial for overall psychosocial development, management of symptoms of comorbid conditions, lowering the chances of relapse, and enhancing adherence to other interventions (e.g., pharmacological treatments) (23). In young people with major depression, a large network meta-analysis found no clear superiority of pharmacological over psychosocial interventions or their combination, but the latest have fewer side effects (24). Current treatment guidelines recommend a combination of psychopharmacological and psychosocial interventions for young people with BD, with an emphasis on ensuring access to structured psychological interventions (21, 25). More research is needed to disentangle which psychosocial interventions may be most beneficial for young people with mood disorders, and to develop stratified and mechanism-based approaches to personalised care (26).

Adolescence is a critical developmental period during which sleep-wake schedules, peer relationships, school demands, family routines, digital activity and autonomy change rapidly (13, 27–30). In unipolar depression, sleep disturbance, reduced activity, irregular routines and social withdrawal may maintain symptoms or impede recovery (31). In bipolar disorder (BD), disruption of social and circadian rhythms is more specifically embedded in models of mood instability and recurrence, with particular clinical concern regarding sleep loss and emerging activation or mania (32, 33). Thus, the two diagnostic groups should not be viewed as mechanistically homogeneous. Nevertheless, irregular sleep-wake timing, reduced daytime structure and unstable daily routines represent clinically relevant behavioural targets across both conditions. Contemporary digital behaviours, including late-night social media and smartphone use, may themselves act as rhythm disruptors by displacing sleep, altering light exposure and weakening regular social time cues (13, 28–30). Existing interventions such as cognitive behavioural therapy often focus primarily on thoughts, emotions or interpersonal stress (22, 34) and may less directly target daily routines, sleep-wake timing, activity patterns and social rhythms. A brief rhythm-focused intervention may therefore be relevant across diagnoses when formulation, risk monitoring and goals are individualised rather than assuming identical illness mechanisms.

Social Rhythm Therapy (SRT), adapted from Interpersonal and Social Rhythm Therapy (IPSRT), provides a framework for targeting these shared behavioural processes. IPSRT combines interpersonal therapy with stabilisation of social rhythms and daily routines and is grounded in social zeitgeber and chronobiological models (32, 33). SRT focuses on the rhythm-stabilising components, including sleep-wake schedules, mealtimes, school or daily activities, physical activity, social contact and evening routines. In STAR-D, these transdiagnostic behavioural targets were retained, but the clinical formulation was diagnosis-sensitive: for unipolar depression, emphasis could include behavioural activation, reversal of withdrawal and restoration of daytime structure; for BD, emphasis additionally included protection against sleep disruption, monitoring for activation or manic symptoms and relapse-prevention planning. The protocol was therefore uniform in its core rhythm-stabilising principles but individually tailored in goals, pacing, risk assessment and clinical application.

In adults, IPSRT has demonstrated efficacy in reducing relapse, extending time to recurrence and improving functioning (32, 35). Importantly, the adult maintenance trial reported that earlier achievement of regular social rhythms was associated with a longer time without a new affective episode, providing established mechanistic evidence that rhythm regularity may contribute to clinical benefit (32). Studies also suggest that IPSRT can reduce symptom severity and improve functioning in adults with bipolar-spectrum disorders (36–38). Rhythm-focused approaches have also been explored in adults with major depressive disorder, including face-to-face and telephone-delivered SRT or IPSRT (39, 40). These findings provide a theoretical and empirical basis for testing rhythm regulation as a candidate mechanism in adolescents, while recognising that mediation must be demonstrated rather than assumed in this age group.

The adolescent evidence base is smaller but directly relevant. Adaptations for adolescents in the United States (US), including IPSRT-A (for adolescents), have sought to account for developmental factors such as circadian phase delay, school timetables, family involvement, digital supports, heightened sensitivity to social influences and the growing need for autonomy (41). Hlastala et al. adapted IPSRT for adolescents with BD and reported high feasibility and acceptability, with improvements in mood symptoms, functioning and treatment satisfaction (41). Goldstein et al. piloted IPSRT in adolescents at familial risk for bipolar disorder, supporting its potential as an early-intervention approach (42). Inder et al. conducted a randomised controlled trial of IPSRT in young people with BD and found improvements in depressive symptoms, manic symptoms and social functioning in both IPSRT and specialist supportive care arms (43). More recently, Sankar et al. showed that predominantly telehealth-delivered SRT was feasible and acceptable for young people with BD, with improvements in social rhythm regularity, mood symptoms and suicide propensity (44). Kim et al. further supported the relevance of daily rhythm regularisation in young people, suggesting potential effects on emotion-regulation circuitry, symptoms and suicide risk (45).

Despite this promising evidence, important gaps remain. Most previous work has focused on adult-derived protocols, research-led settings and BD rather than unipolar depression, with much of the adolescent evidence originating in the United States. Adult-derived protocols may be difficult for young people to follow when they do not account for school timetables, dependence on family routines, developmental changes in autonomy, social and digital life, and the burden of repeated self-monitoring. Limited direct involvement of adolescents in protocol development may therefore reduce relevance, adherence and sustainability. Co-development can enhance acceptability and implementation fit (46), and international guidance recommends treating young people as partners rather than solely as research participants (47). The UK Medical Research Council framework for complex interventions similarly emphasises shared development, participatory design and iterative refinement (48).

The present study addressed these gaps by co-developing an adolescent-adapted form of SRT with young people and clinicians and piloting it within routine NHS CAMHS. The study aimed to: (a) co-design the STAR-D protocol in collaboration with young people and clinicians; (b) examine its feasibility and acceptability for adolescents with unipolar and bipolar depression; (c) identify clinical, participant-related and contextual barriers and facilitators; and (d) generate recommendations for optimisation and future larger-scale trials. The study was designed to provide preliminary feasibility and acceptability evidence, not to evaluate effectiveness or establish transferability beyond the participating London service.

## Methods

### Study design, setting and governance

This study used a two-phase mixed-methods feasibility design to co-produce, adapt and pilot Social Therapy for Adolescent Rhythms in Depression (STAR-D), the specific adolescent-adapted SRT protocol evaluated in this study. We use “SRT” when referring to the general therapeutic framework and “STAR-D” when referring to the co-designed intervention tested here. Phase 1 involved co-design and intervention adaptation with the Lambeth Young People's Advisory Group (YPAG), a forum of young people with lived experience, and clinicians. Phase 2 was a pilot case series evaluating feasibility, acceptability and exploratory clinical outcomes among adolescents receiving care within Lambeth CAMHS, South London and Maudsley NHS Foundation Trust.

Qualitative reporting was guided by the COREQ checklist (summary in Table 1; full checklist in Supplementary eTable 1) (49). The intervention was described using the Template for Intervention Description and Replication (TIDieR) checklist (50). The project was reviewed and approved as a service-development and feasibility project by the South London and Maudsley NHS Foundation Trust CAMHS Governance Board (ID 493); formal NHS Research Ethics Committee approval was not required under the applicable local governance pathway. All participating young people and caregivers provided informed consent before participation.

**Table 1:** Summary of COREQ domains for qualitative reporting.

**Domain 1: Research team and reflexivity**
*Personal Characteristics:* Phase 1 feedback/interviews and Phase 2 post-intervention feedback were conducted by members of the research/clinical team, including CA, GSP and ZK. GSP and CA were medically qualified clinicians with PhDs, and ZK was an MSc student. GSP was a senior clinical lecturer, CA a post-doctoral researcher, and ZK an MSc student. The team included both male and female researchers/clinicians. Relevant clinical, research and qualitative experience informed data collection and interpretation.
*Relationship with participants:* GSP was the consultant psychiatrist for some participants. For Phase 1, young people were engaged through the Lambeth YPAG and co-production activities. For Phase 2, participants received SRT from researchers/clinicians involved in the study. Participants were informed about the study transparently, including the role of the researchers and clinicians involved.
**Domain 2: Study design**
*Theoretical framework:* The study used a two-phase mixed-methods feasibility design. Phase 1 used co-production methods to adapt SRT for adolescents. Phase 2 used a pilot case-series design to assess feasibility, acceptability and exploratory clinical outcomes*.*
*Participant selection:* Phase 1 used convenience sampling of young people aged 12–18 years with sleep and mood difficulties, including Lambeth YPAG input, and clinician feedback. Phase 2 participants were recruited from Lambeth CAMHS clinical pathways. Young people were approached through CAMHS/co-production routes and routine clinical or research contacts. Phase 1 included 11 young people and six clinicians. Phase 2 included six participants. One young person did not participate because they preferred exclusive pharmacological management.
*Setting:* Phase 1 data were collected through online questionnaire feedback, Lambeth YPAG involvement and clinician feedback. Phase 2 was conducted within Lambeth Child and Adolescent Mental Health Services (CAMHS), South London and Maudsley NHS Foundation Trust, as part of routine clinical care
*Data collection:* Phase 1 data included online questionnaire responses, YPAG discussion/interview material and clinician feedback. Phase 2 data included demographic and clinical information, feasibility indicators, post-intervention acceptability feedback and exploratory clinical outcome measures.
**Domain 3: Analysis and findings**
*Data analysis:* Phase 1 qualitative data were examined descriptively and organised into themes relevant to intervention adaptation. Phase 2 qualitative feedback was analysed descriptively, focusing on feasibility, acceptability, perceived usefulness and burden. Quantitative clinical outcomes were analysed descriptively only, with no inferential statistical testing because of the small pilot case-series design.
*Reporting:* Findings are reported sequentially. First, Phase 1 co-production findings are presented, showing how young people's and clinicians’ feedback informed adaptation of SRT. Second, Phase 2 findings are presented, including feasibility, acceptability and exploratory clinical outcome trajectories.

### Phase 1: co-production and adaptation of SRT

A co-production and adaptation process was undertaken between October 2024 and July 2025, to ensure that SRT was developmentally appropriate, clinically relevant and feasible for adolescents in our context. The aim was to adapt the intervention while preserving its core therapeutic mechanism: stabilising daily routines, sleep–wake patterns, activity and social rhythms to support mood regulation.

The co-production process drew on three sources of feedback. First, an online feedback questionnaire containing structured, semi-structured and open-ended items was completed by 10 young people aged 13–18 years from the general population who reported difficulties with sleep, mood regulation and maintaining daily routines (see Supplementary eFigure 1 for recruitment flyer and Supplementary eFigure 2 for feedback form). Study information was presented during team meetings, and recruitment flyers were distributed within the service.

The questionnaire explored intervention content, preferred delivery format, potential barriers and facilitators to engagement, and experiences of daily routines. Second, the intervention was presented by CA and GSP to the Lambeth YPAG. To obtain more detailed feedback, one YPAG member completed an in-depth semi-structured interview, which explored experiences of daily routines, perceived challenges, and suggestions for making the intervention more relevant, accessible and engaging for adolescents. Notes from this interview were used to refine the intervention protocol, and the young person agreed for anonymised quotations to be used. Third, six clinicians from Lambeth CAMHS, including author and non-author contributors, reviewed the initial protocol and provided feedback on feasibility, clinical applicability and fit with routine CAMHS delivery, from January 2025 to March 2025. Clinician feedback was incorporated to ensure that the adapted protocol aligned with service context and addressed the needs of adolescents with mood disorders. Overall, Phase 1 therefore included 11 young people and six clinicians contributing individual feedback. Co-authors from this manuscript have also made suggestions informally.

Feedback from young people, the YPAG interview and clinicians was reviewed and organised descriptively into key themes. The authors then reviewed these themes collaboratively to identify adaptation priorities. This process informed the adapted SRT protocol we then used. The final adapted intervention is summarised using the TIDieR framework -see Table 2 (50).

**Table 2:** TIDieR description of the adolescent-adapted social rhythm therapy (STAR-D).

Item category	Description
Brief name	STAR-D: Social Therapy for Adolescent Rhythms in Depression, a co-designed, rhythm-focused psychological intervention for adolescents with depressive disorder.
Why	Adolescents with bipolar disorder and major depressive disorder commonly experience disrupted sleep–wake timing, irregular daily routines, reduced activity, social withdrawal and mood instability. SRT aims to stabilise daily social, sleep and activity rhythms, thereby supporting mood regulation, functioning and relapse prevention. The intervention was adapted because existing rhythm-focused therapies were largely developed for adults and did not fully reflect adolescents’ developmental needs, school routines, autonomy, family context, social media use and preferences for flexible support.
What: Materials	The intervention used our adolescent-adapted SRT protocol and supporting materials, including: *1. Participant routine/social rhythm monitoring forms:* used to track sleep, waking time, meals, activity, social contact, mood and key daily routines. *2. Goal-setting worksheets:* used to identify personalised routine-stabilisation goals using SMART principles. *3. Psychoeducation materials:* explaining links between mood, sleep, routines, activity, social rhythms and relapse risk. *4. Session summaries/reminders:* used where appropriate to help participants remember key content between sessions. 5*. Clinician protocol/manual:* outlining session structure, therapeutic aims, suggested questions, monitoring procedures, safety considerations and strategies for adapting content to adolescents. 6*. Feedback forms:* used post-intervention to assess acceptability, satisfaction, perceived usefulness and burden.
What: Procedures	The intervention followed a structured but flexible therapeutic process. The first session focused on engagement, rapport building, understanding the young person's interests and daily routine, and collaboratively identifying treatment goals. Subsequent sessions reviewed routine and mood monitoring, identified rhythm disruptors, explored links between sleep, routines, activity and mood, and supported gradual changes to daily patterns. Therapeutic strategies included psychoeducation, collaborative problem-solving, activity scheduling, sleep–wake regularisation, planning around school/weekend transitions, managing disruptions, and reviewing progress. Sessions also included recap and consolidation of learning.
Who: Provided	Sessions were delivered by CA or GSP, both consultant child and adolescent psychiatrists with extensive clinical and research experience. Clinical safety and individual adaptation were supported through routine CAMHS governance and multidisciplinary care.
How	SRT was delivered individually, face-to-face, in a clinical setting. Sessions were collaborative and youth-centred, with flexibility in scheduling and pacing. The intervention was delivered one-to-one between clinician and participant, with parent/carer involvement considered where clinically appropriate or requested, but not required as a core component.
Where	The pilot was delivered within Lambeth Child and Adolescent Mental Health Services, South London and Maudsley NHS Foundation Trust, as part of a service development project for adolescents receiving routine CAMHS care. Participants were recruited from Lambeth CAMHS following discussion with clinical teams and distribution of recruitment materials.
When and how much	The intervention was carried out in a clinical setting (Lambeth CAMHS) and consisted of 8 sessions, each lasting 60 min.
Tailoring	The intervention was tailored to each young person's routines, goals, symptoms, school/college timetable, family context, sleep pattern, activity level, social life and motivation. Participants selected personally meaningful rhythm goals, such as improving sleep timing, getting up more consistently, managing weekend disruption, increasing daytime activity, or creating more predictable routines around school, meals, social contact and evening phone use. Flexibility in session timing, pacing and order of topics was used to support engagement.
Modifications	Co-design feedback led to adolescent-friendly framing, additional rapport building, examples based on school, social and family life, flexible appointments, and an eight-session rather than 12-session format. Monitoring was simplified, and future versions should test lower-burden paper or digital approaches.
How well: Planned	Feasibility and acceptability were planned to be assessed through referral, uptake, retention, completion, dropout, feedback and clinician observations. Between-session monitoring was used therapeutically; prospective quantitative adherence capture was not built into this service-development pilot. Clinical outcomes were descriptive.
How well: Actual	Seven young people were referred, six started and five completed STAR-D. Participants valued the collaborative and flexible approach, but daily monitoring was experienced as burdensome. Monitoring-form completion and missingness were not quantified because forms were not systematically retained as research data. Clinical outcomes were analysed descriptively.

### Phase 2: pilot feasibility case series

Phase 2 piloted STAR-D in adolescents with unipolar or bipolar depression receiving clinical care in Lambeth CAMHS. Participants were recruited through Tier 3 specialist CAMHS or the crisis service (51). Before starting the intervention, eligible participants completed a demographic questionnaire documenting age, sex, gender identity, ethnicity and co-occurring conditions. Inclusion criteria were: (a) age 12–18 years; (b) a clinical diagnosis of major depressive disorder, bipolar I disorder or bipolar II disorder, defined using ICD-10 criteria and recorded in the clinical record; and (c) current depressive symptoms confirmed clinically and quantified using the Montgomery-Asberg Depression Rating Scale (MADRS) at baseline. No minimum MADRS cut-off was prespecified because this feasibility pilot recruited clinically diagnosed young people already receiving specialist care; the MADRS was used to describe symptom severity and change, not to establish the diagnosis or determine eligibility by a numerical threshold. Exclusion criteria were cognitive impairment, active suicidal ideation with intent or plan requiring urgent higher-intensity care, and active psychotic features.

STAR-D consisted of eight 60-minute weekly one-to-one sessions delivered in Lambeth CAMHS by CA or GSP, both consultant psychiatrists with extensive clinical and research experience. The intervention followed a structured but flexible protocol and was tailored to diagnosis, developmental stage, clinical risk and individual goals. Participants were asked to complete one brief paper routine-monitoring entry each day between sessions. The adapted Social Rhythm Metric (SRM) recorded the timing of key daily events, including getting out of bed, meals, school or another main daytime activity, social contact and bedtime, alongside mood and notable rhythm disruptors (52). Entries were reviewed collaboratively at the next session to identify patterns and set realistic rhythm-stabilisation goals. The form was simplified from the original SRM for adolescent use; a reproducible template is provided in Supplementary eTable 2. Subsequent sessions reviewed progress, barriers and facilitators, adjusted routines or behavioural plans, and considered links between mood fluctuations and daily rhythms. Core techniques included psychoeducation, collaborative problem-solving, behavioural activation and structured activity scheduling.

### Outcomes and analysis

The primary outcomes were feasibility and acceptability. Feasibility was assessed through referral, uptake, retention, completion and dropout. Acceptability was assessed through qualitative post-intervention feedback, including satisfaction with content and delivery, perceived usefulness and experience of between-session monitoring. Secondary outcomes were exploratory clinical outcomes assessed at baseline (week 0), mid-intervention (week 4) and end of intervention (week 8): MADRS depressive symptom severity (53), Young Mania Rating Scale (YMRS) manic symptom severity (54), and Children's Global Assessment Scale (CGAS) global functioning (55). The Global Assessment of Functioning (GAF) (56) was listed in the initial draft in error and was not administered as a separate outcome; the age-appropriate CGAS was used for global functioning.

Phase 1 qualitative material was examined descriptively and organised into themes. Phase 2 feedback was analysed descriptively, focusing on feasibility, acceptability, perceived usefulness and burden; NVivo version 15 supported coding (57). Routine-monitoring forms were used as therapeutic aids and were not systematically archived or coded as research data. Consequently, quantitative log-completion percentages and item-level missingness could not be calculated; this limitation is reported explicitly. Given the small exploratory case-series sample, clinical outcomes were analysed descriptively and presented using individual trajectories and exact scores, without inferential testing.

Results are presented sequentially. Phase 1 findings describe five areas relevant to adaptation: adolescents’ understanding of SRT and links among routines, sleep and mood; routine contexts and disruptors; preferences for intervention structure, delivery and monitoring; barriers and facilitators; and suggested adaptations. These themes informed STAR-D. Phase 2 findings then describe feasibility, acceptability and exploratory clinical trajectories.

## Results

### Phase 1: co-production and adaptation of SRT

#### Understanding of SRT and perceived links between routines, sleep and mood

Young people varied in their initial understanding of the term “Social Rhythm Therapy”. In the online questionnaire, several participants initially associated the term with music, rhythm, or social activities, while others understood it as a therapy focused on daily routines and mood regulation. This suggested that the intervention would need to be clearly introduced using accessible, adolescent-friendly language.

Across the questionnaire and YPAG feedback, young people recognised that routines, sleep and mood were connected. In the online questionnaire, respondents agreed that sleep and routines affect their mood. Participants described how disrupted routines could lead to low energy, reduced motivation, irritability, poorer concentration, reduced social engagement and difficulties with schoolwork. A YPAG interviewee similarly described how staying up late at weekends could have a “knock-on effect” over several days, affecting mood, motivation and the ability to re-establish routine. “*If you spend every Saturday out of routine and stay up until 4 am, you can feel out of balance for days afterwards.”* This theme informed the intervention by highlighting the need to explain SRT as a practical approach to understanding and improving the links between routines, sleep, activity and mood, rather than as a rigid or prescriptive programme.

#### Daily routine contexts and rhythm disruptors

Young people's routines were strongly shaped by external commitments, especially school and college timetables, but also family context (e.g., scheduling of mealtimes and bedtime routines). Many questionnaire respondents described education as providing structure through fixed wake times, travel, lessons and scheduled activities. Our interviewee described having different routines for college and non-college days, with the college timetable acting as an anchor for daily rhythm. “*Days without much routine are particularly difficult, especially when I am not in college”.*

**Figure 1:**
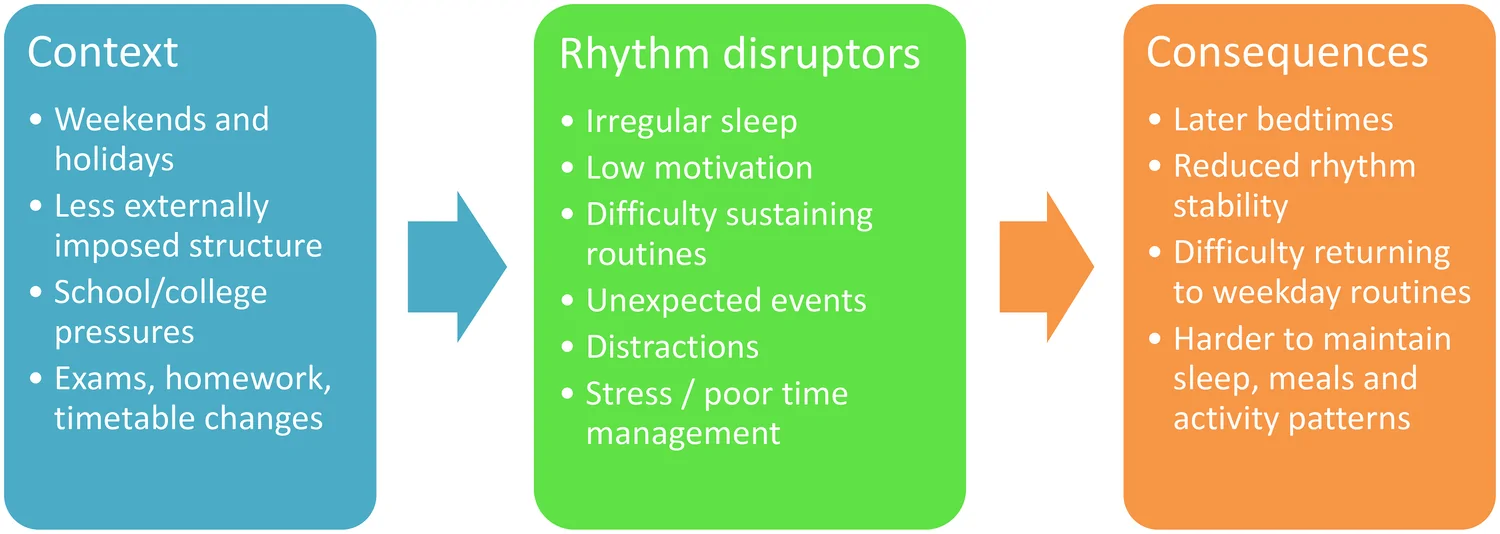
Contexts, rhythm disruptors and perceived consequences identified during phase 1 co-production.

However, young people also described several rhythm disruptors. These included irregular sleep, low motivation, difficulty sustaining routines, unexpected events, distractions, stress and poor time management. Weekends and holidays were described as particularly challenging because externally imposed structure was reduced, often leading to later bedtimes and difficulty returning to weekday routines (Figure 1). This pattern is consistent with social jetlag, whereby discrepancies between weekday and free-day sleep timing may disrupt circadian regularity and contribute to poorer mood (58). These themes informed the adaptation of SRT by emphasising the importance of mapping each young person's actual context, including school or college structure, weekends, holidays, social activities, family expectations and individual rhythm disruptors. It also supported the importance of making routine goals personalised and realistic rather than standardised.

#### Preferences for intervention structure, delivery and monitoring

Young people expressed a preference for a flexible and practical intervention. Questionnaire respondents suggested different possible session frequencies, including weekly, fortnightly or as-needed sessions. Preferences for delivery format were mixed, with some favouring in-person delivery, and others suggesting a hybrid approach. Clinicians similarly supported a structured intervention but emphasised the need for flexibility in session length, pacing and module order.

Routine tracking was seen as potentially useful, but young people also highlighted that monitoring could become burdensome. In the online questionnaire, 90% of respondents indicated a preference for daily reminders (e.g., digital reminders). This theme informed the intervention by supporting flexible scheduling, adolescent-relevant examples, optional use of reminders (particularly on the phone) or written summaries, and attention to the burden of routine monitoring.

#### Barriers and facilitators to engagement

Young people identified several potential barriers to engaging with SRT. These included low motivation, uncertainty about whether the therapy would help, reluctance to share personal information with family or friends, competing commitments and difficulty remembering session content between appointments. The YPAG interviewee suggested that sessions spaced too far apart could make it harder to maintain progress, particularly early in therapy. “*Having sessions close together and reviewing things weekly would help, because there is a learning curve before it becomes second nature”.*

Facilitators included clear schedules, reminders, support from trusted adults or friends, milestone recognition, personalised feedback and a collaborative therapeutic style. Clinicians also highlighted service-level facilitators and barriers, including the importance of trained staff, integration into existing CAMHS pathways and flexibility around appointment timing. “*It needs to be personalised, because everyone's daily routine is personal”.* This theme informed the intervention by emphasising rapport building, collaborative goal-setting, session summaries, flexible appointments and a supportive rather than directive therapeutic style.

#### Suggested adaptations to the intervention

**Figure 2:**
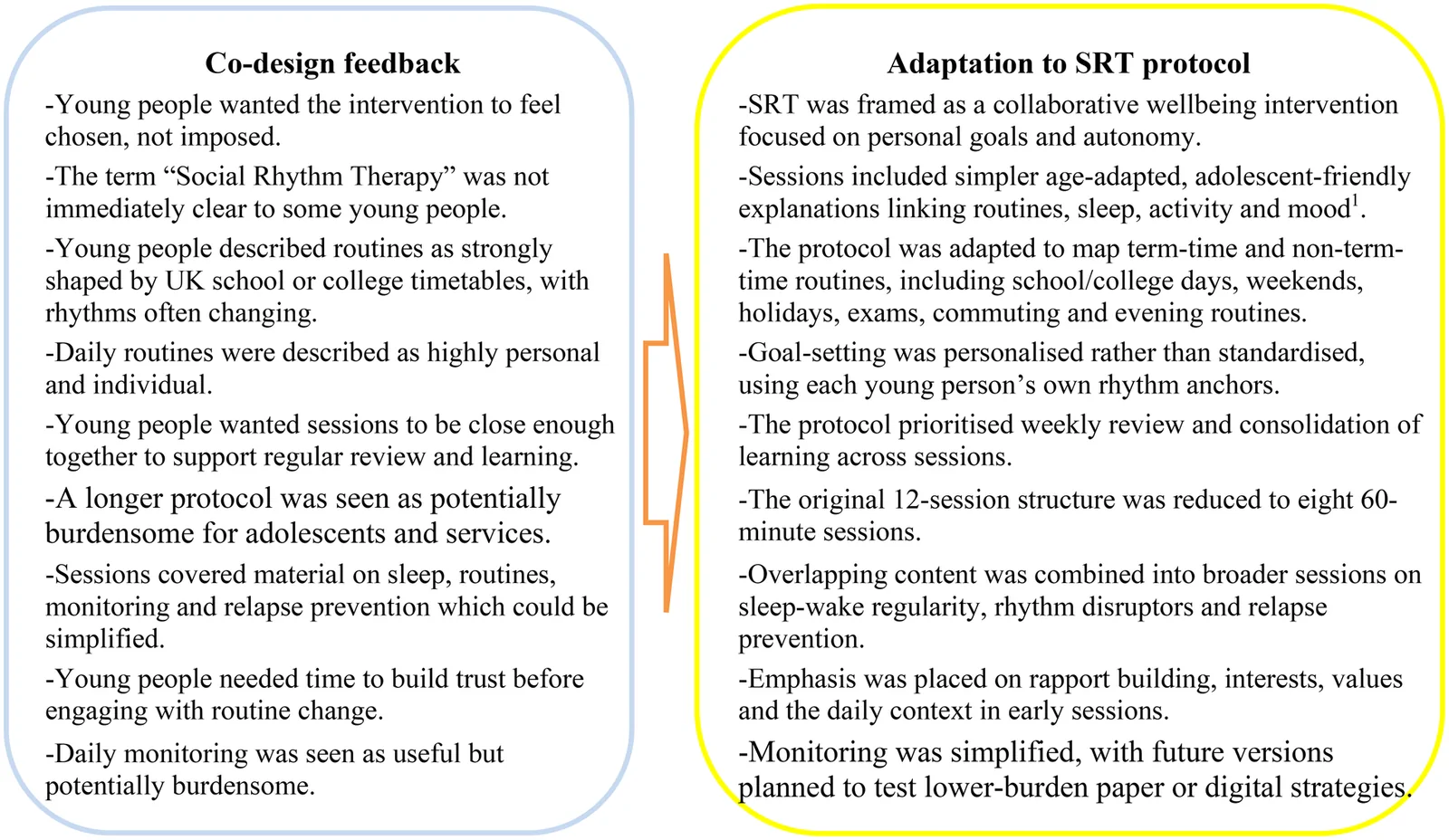
Co-design feedback and corresponding adaptations to the adolescent-adapted SRT protocol. 1 Age-adapted examples illustrated developmental differences in routine disruption. For younger adolescents, explanations focused on concrete daily anchors such as getting up, breakfast, school, after-school activities, shared meals and parent-supported bedtime routines. For older adolescents, examples emphasised greater autonomy, including college timetables, part-time work, independent travel, social plans, screen use, exercise and weekend routines.

Phase 1 feedback led to several adaptations to STAR-D. SRT was framed as a positive and collaborative approach to wellbeing rather than an imposed routine; additional time was allocated to rapport building; examples reflected school or college, weekends, sleep timing, social and digital activities, family context and motivation; and sessions were delivered flexibly. The burden of daily monitoring was recognised as an important issue. Individual strategies, such as setting a recurring phone reminder during a session, were suggested, alongside simplified or lower-burden digital options. Young people emphasised explicitly linking routines to mood, energy and functioning and incorporating practical and motivational supports. Figure 2 summarises the co-design feedback and corresponding adaptations, and Table 3 presents the eight-session protocol.

**Table 3:** Structure and content of the eight-session adolescent-adapted social rhythm therapy protocol.

*Session 1 — Engagement, formulation and daily rhythm map* Build rapport; understand the young person's mood history, interests, school/family context and current routine. Introduce SRT as a collaborative wellbeing intervention, not an imposed routine. Start mapping sleep, meals, school, activity, social contact and mood.
*Session 2 — Social rhythms, mood and monitoring* Explain the link between routines, social zeitgebers, sleep-wake timing and mood. Introduce the Social Rhythm Metric/routine tracker. Identify the young person's main rhythm disruptors and one or two realistic rhythm anchors.
*Session 3 — Sleep-wake regularity I: wake time and evening routine* Focus on sleep timing, bedtime habits, wake time, circadian phase delay in adolescence, and evening routines. Agree on small goals around consistent wake time, wind-down routine, screens/phone use and sleep preparation.
*Session 4 — Sleep-wake regularity II: weekends, school and disruptions* Work on weekend routine shifts, school/college schedules, holidays, exams, social media, naps, light exposure and practical barriers. Develop strategies for returning to routine after disruption.
*Session 5 — Activity, meals and behavioural rhythm goals* Target daytime activity, physical activity, meals, social contact and behavioural activation. Use SMART goals to stabilise daily routines linked to mood, energy, concentration and functioning.
*Session 6 — Social rhythms, relationships and family/school context* Explore how family routines, peer relationships, school demands, interpersonal stress and support networks affect rhythms. Consider parent/carer or school involvement only where appropriate and acceptable to the young person.
*Session 7 — Managing rhythm disruption and relapse prevention* Identify predictable and unpredictable rhythm disruptors: stress, illness, holidays, conflict, low motivation, mood changes, medication changes, exams or social events. Create a personal early-warning and routine-recovery plan.
*Session 8 — Consolidation, maintenance and ending* Review progress, what helped, what was difficult, and which rhythm goals are realistic to continue. Create a maintenance plan, relapse-prevention plan and personalised “what to do when routines slip” strategy.

### Phase 2: pilot feasibility and acceptability case series

#### Participant characteristics

Seven young people were referred and six accepted and started STAR-D. Participants were aged 13–17 years; four identified as male and two as female. Three had bipolar II disorder, two had major depressive disorder and one had bipolar I disorder. Ethnic backgrounds were White British (*n* = 2), White Other (*n* = 1), Black British (*n* = 1), Hispanic (*n* = 1) and Mixed Race (*n* = 1). Because this was a very small single-service sample with uncommon diagnostic-demographic combinations, these characteristics are reported in aggregate to reduce deductive disclosure risk. Exact de-identified clinical scores for the five completers are provided by participant in Table 4.

**Table 4:** Participant-level exploratory clinical outcome scores among the five completers.

Participant	CGAS (W0/W4/W8)	MADRS (W0/W4/W8)	YMRS (W0/W4/W8)
1	10/7/33	31/45/22	6/3/3
2	50/75/75	28/5/3	7/1/9
3	65/71/74	16/20/25	27/11/15
4	75/80/80	20/13/14	13/10/9
5	56/50/55	36/38/32	4/4/5

Values are shown for week 0/week 4/week 8.

CGAS, Children's Global Assessment Scale; MADRS, Montgomery-Asberg Depression Rating Scale; YMRS, Young Mania Rating Scale.

#### Feasibility and acceptability

Feasibility outcomes indicated good initial uptake and completion. Of seven young people referred, six accepted and started STAR-D (85.7%); five of the six starters completed all eight sessions (83.3%). One participant discontinued after three sessions. The reason for discontinuation was not documented in the feasibility dataset and therefore cannot be classified as clinical deterioration, scheduling difficulty or intervention burden. STAR-D was delivered flexibly over 8–12 weeks according to availability and clinical need, face-to-face and individually by GSP or CA within Lambeth CAMHS.

Acceptability feedback suggested that the intervention was experienced as supportive, collaborative and accessible. Participants valued feeling listened to, taken seriously and informed about available support. The structured session format and focus on personalised goals appeared to support engagement. Participants also described the service as convenient and felt that professionals worked collaboratively to support them.

Participants were asked to make one brief paper entry each day, recording the timing of key routine anchors and mood, and to review these entries during weekly sessions. Qualitative feedback indicated that this daily monitoring could feel burdensome, consistent with Phase 1 feedback. Because the forms were used as clinical tools and were not systematically retained as research data, log-completion percentages and missing-item frequencies are unavailable. This limits the empirical precision with which monitoring adherence can be described. Future versions should preserve collaborative awareness of routines while testing briefer forms, event-contingent entries, digital reminders or carefully co-designed passive measures.

Key suggestions integrated from young people’s feedback included: (a) offering flexibility in session day and time to fit with other activities; (b) providing written summaries or reminders between sessions; (c) allowing an extended initial period for rapport building; (d) supporting young people to identify realistic routine goals; and (e) involving parents, carers or other relevant supports where appropriate and acceptable to the young person.

#### Exploratory clinical outcomes

**Figure 3:**
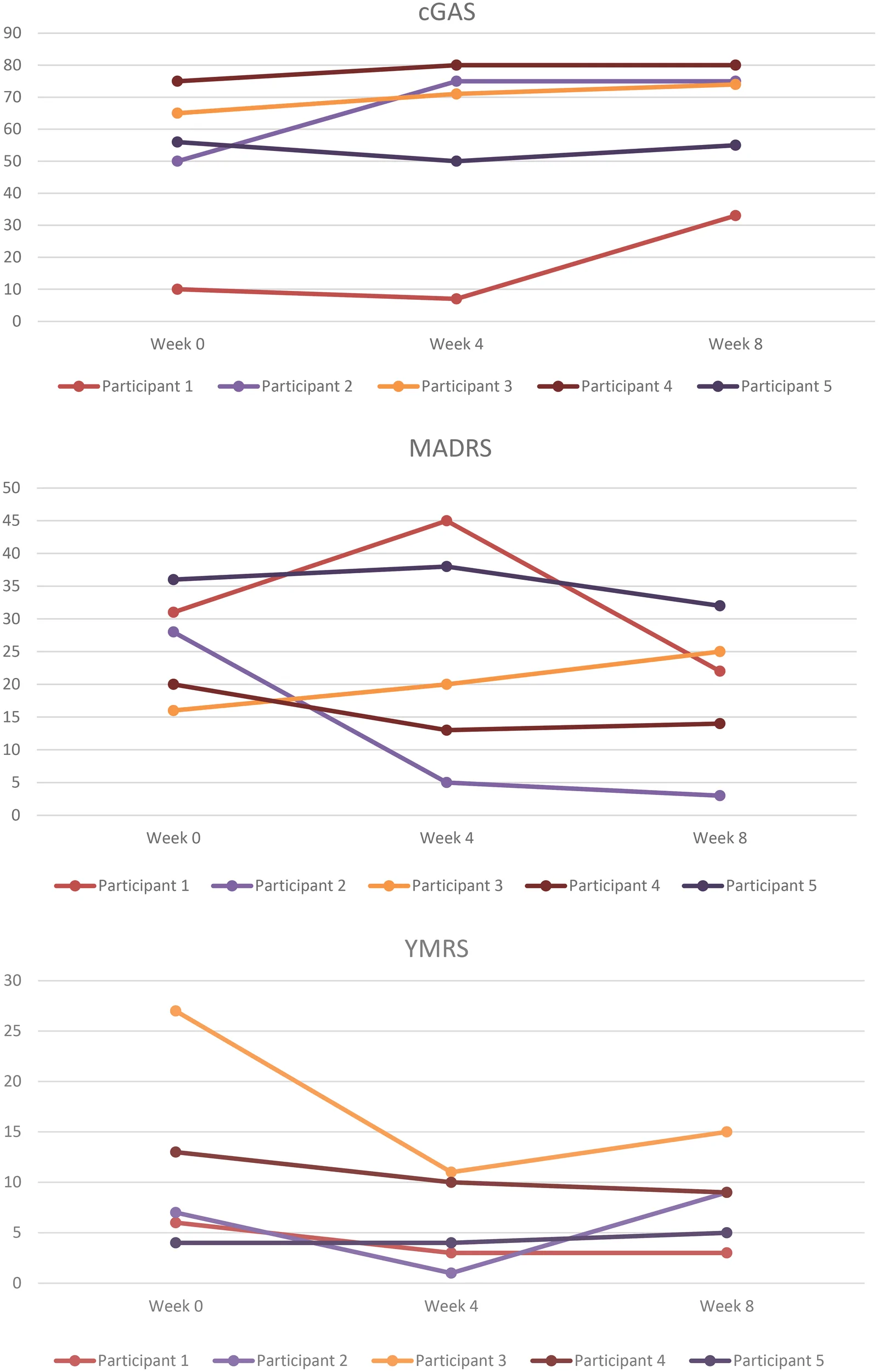
Exploratory clinical trajectories during adolescent-adapted social rhythm therapy. **(A)** Functioning measured using the Children's Global Assessment Scale (CGAS), where higher scores indicate better functioning. **(B)** Depression severity measured using the Montgomery–Åsberg Depression Rating Scale (MADRS), where higher scores indicate greater depressive symptom severity. **(C)** Mania severity measured using the Young Mania Rating Scale (YMRS), where higher scores indicate greater manic symptom severity.

Among the five completers with repeated outcome data, trajectories were variable. For example, one participant's CGAS score increased from 10 at week 0 to 33 at week 8, while another remained stable at 75. MADRS trajectories included both decreases and increases; one participant decreased from 28 to 3, whereas another increased from 16 to 25 by week 8. YMRS scores were generally low or decreased, although variability was observed. See Table 4 and Figure 3 for participant-level baseline characteristics and clinical scores at weeks 0, 4 and 8. These outcomes are descriptive only and should not be interpreted as evidence of efficacy.

## Discussion

The present study piloted STAR-D, a developmentally adapted SRT protocol created through youth and clinician co-design for adolescents with unipolar and bipolar depression. Seven young people were referred, six started and five completed all eight sessions. Feedback suggested that the intervention was collaborative, supportive and accessible, while daily monitoring required further refinement. These findings provide preliminary evidence that a brief rhythm-focused intervention can be delivered in routine CAMHS; they do not establish effectiveness, diagnostic equivalence or scalability.

The rationale for rhythm stabilisation differs partly by diagnosis. In unipolar depression, restoration of daytime structure, activity and social engagement may support recovery from withdrawal and inactivity. In BD, regular sleep-wake and social rhythms are more directly linked to relapse models, and intervention requires additional vigilance for activation, reduced need for sleep and manic symptoms (32, 33). Prior adolescent SRT and IPSRT studies have mainly involved BD or familial risk (41–45), whereas adult work also supports feasibility in major depressive disorder (39, 40). STAR-D therefore used shared behavioural targets but diagnosis-sensitive formulations and safety monitoring. The present sample is far too small to compare clinical trajectories by diagnosis, and no diagnosis-specific benefit can be inferred.

This study extends the literature by providing preliminary feasibility data on a brief, co-designed SRT protocol delivered within routine CAMHS and offered to adolescents with both bipolar and unipolar depression. It may help inform the design of larger controlled studies addressing the limited evidence base for developmentally appropriate rhythm-focused interventions. Claims regarding clinical benefit or scalability require confirmation in adequately powered trials delivered by a broader range of practitioners.

Another key contribution of this study is the comprehensive co-design process, consistent with recommendations to involve patients and the public in intervention development and adaptation (46), and with youth mental health co-design work showing that lived-experience input can improve relevance, acceptability and implementation fit (59). Young people's feedback highlighted that the intervention needed to be explained using accessible, adolescent-friendly language. Participants readily understood the link between sleep, routines and mood when this was framed practically, for example around school days, weekends, holidays, motivation, energy and concentration. Acceptability of rhythm-focused intervention may depend not only on its therapeutic content but also on how it is introduced. Presenting routines as a positive, autonomous choice appeared particularly important. This aligns with self-determination theory-informed approaches, which emphasise that health-related behaviour change is more likely to be sustained when it supports autonomy, personal relevance and intrinsic motivation (60). These findings are consistent with recent WHO guidance on youth co-creation, emphasising meaningful collaboration, accessibility and shared decision-making, as well as intervention frameworks that stress stakeholder engagement and contextual adaptation (47, 48).

The co-design findings underscore the developmental specificity of adolescence. School and college timetables acted as external anchors, whereas weekends, holidays, exams, changing family routines and social media could disrupt rhythms. Contemporary digital activity may modify social zeitgebers through late-night engagement, variable social contact, light exposure and displacement of sleep, extending rather than replacing the foundational social zeitgeber model (13, 28–30, 33, 45, 61, 62). STAR-D therefore addressed phone and screen use within individual rhythm maps and evening routines. Adolescents also have less control than adults over family and school schedules, supporting collaborative and developmentally tailored rather than prescriptive rhythm goals.

Daily monitoring was the clearest optimisation target. The SRM is intended to increase awareness of links among routines, rhythms and mood (33, 52), but repeated paper recording may add cognitive and motivational burden. Smartphone-based ecological momentary assessment and experience-sampling approaches can capture experiences closer to their occurrence and reduce dependence on retrospective recall, while digital reminders and passive sensing may reduce some manual entry requirements (62–64). However, digital monitoring is not inherently low-burden and introduces privacy, acceptability and surveillance concerns. Future STAR-D monitoring should therefore be brief, optional where clinically appropriate, developmentally co-designed and evaluated empirically for completion, missingness and acceptability.

The study also informs future mechanistic trials. Adult IPSRT evidence already supports social-rhythm regularity as a plausible mechanism: earlier achievement of regular rhythms was associated with longer time without recurrence (32). The unanswered question is whether this mechanism operates in adolescents with unipolar depression, BD or both. Future trials should combine subjective rhythm measures and sleep diaries with actigraphy or wearable measures of sleep timing, regularity, activity and rest-activity stability (52, 62, 64, 65). Mediation analyses should test whether changes in these measures precede changes in depressive symptoms, manic symptoms, functioning and quality of life (66). Such trials should be powered to examine diagnostic moderation rather than presuming identical pathways.

Implementation conclusions must remain cautious. STAR-D was delivered to a small, selected sample by two senior consultant psychiatrists, which may have enhanced engagement and retention. Before scalability or task-sharing can be claimed, future studies should assess delivery by clinicians with varied professional backgrounds, using standardised training, fidelity assessment and supervision. They should also determine referral criteria, protected time for reviewing monitoring data, parent or school involvement, intervention costs and service fit. The present London pilot does not establish cultural or international transferability; any evaluation in other communities or resource settings should begin with local co-design and a separate assessment of contextual fit.

Several strengths should be noted, including participatory co-design, delivery in routine CAMHS and integration of feasibility indicators with qualitative feedback and exact descriptive trajectories. Important limitations are substantial. First, Phase 1 included general-population adolescents reporting difficulties with sleep, mood regulation and routines, whereas Phase 2 involved clinically diagnosed young people, including referrals from specialist and crisis pathways; this population mismatch may have limited the clinical specificity of some adaptations. Second, the Phase 2 sample was very small, uncontrolled, recruited from one service and diagnostically heterogeneous. Combining unipolar depression and BD improved pragmatic relevance but prevents diagnosis-specific inference and may obscure differences in vulnerability, risk and mechanism. Third, delivery by two senior consultant psychiatrists limits conclusions about acceptability under routine workforce or task-sharing conditions; provider expertise and motivated participants may have contributed to retention and satisfaction. Fourth, authors were involved as clinicians, intervention developers and analysts across both phases. This dual clinician-researcher role may have introduced confirmation and social-desirability biases during co-production, delivery, coding and interpretation. Fifth, the reason for the single dropout and quantitative monitoring adherence were not systematically recorded, and GAF was not administered separately. Outcome data were descriptive, RCADS was not used for follow-up, formal saturation and participant checking were not undertaken, and assessors were not blinded. Without a comparator, clinical changes may reflect concurrent treatment, natural course, regression to the mean, service contact or external events. Quantitative completion of daily monitoring forms were not prospectively collected. Finally, findings from one London CAMHS service cannot establish effectiveness, scalability or cultural transferability.

In conclusion, STAR-D was feasible to deliver and acceptable to most young people in this small routine-CAMHS pilot. The findings support further optimisation of monitoring and delivery procedures and provide a rationale for an adequately powered controlled mechanistic trial. Future studies should test whether rhythm regularisation mediates changes in mood and functioning, evaluate diagnostic moderation, and examine delivery by a broader range of practitioners. The current findings are feasibility and acceptability evidence only, not evidence of effectiveness or transferability.

## Data Availability

The raw data supporting the conclusions of this article can be made available by the authors upon request.
