# Supplementary material for: Co-designing and piloting adolescent-adapted social rhythm therapy for young people with unipolar and bipolar depression: a mixed-methods feasibility case series
Source: Front Child Adolesc Psychiatry. 2026 Aug 28;5:1909726. doi: 10.3389/frcha.2026.1909726 (PMC13562024; doi:10.3389/frcha.2026.1909726)
Supplement: Supplementary file 1 [file Supplementaryfile1.docx]

**SUPPLEMENTARY MATERIAL**

- Supplementary eTable 1. COREQ checklist for qualitative reporting
  - Pages 2-4
- Supplementary eFigure 1. Patient and Public Involvement/co-production recruitment flyer used in Phase 1
  - Page 5
- Supplementary eFigure 2. Young person feedback form used during Phase 1 co-production
  - Page 6-7
- Supplementary eFigure 3. Recruitment flyer for the Social Rhythm Therapy pilot case series
  - Page 8
- Supplementary eFigure 4. Weekly Social Rhythm Metric (SRM) form used for daily routine and mood monitoring in the Phase 2 STAR-D pilot
  - Page 9

**eTable 1: COREQ checklist for qualitative reporting**

| **No** | **Item** | **Guide questions/description** |
| --- | --- | --- |
| **Domain 1: Research team and reflexivity** | | |
| Personal Characteristics | |  |
| 1. | Interviewer/facilitator | Phase 1 feedback/interviews and Phase 2 post-intervention feedback were conducted by members of the research/clinical team, including CA, GSP and ZK |
| 2. | Credentials | GSP: MD, PhD; CA: MD, PhD; ZK: MSc |
| 3. | Occupation | GSP: senior clinical lecturer; CA: post-doctoral researcher; ZK: MSc student |
| 4. | Gender | Both females and males. |
| 5. | Experience and training | GSP: MD, PhD; CA: MD, PhD; ZK: MSc |
| Relationship with participants | |  |
| 6. | Relationship established | GSP was the consultant psychiatrist for some of the patients. |
| 7. | Participant knowledge of the interviewer | For Phase 1, young people were engaged through Lambeth YPAG/co-production activities. For Phase 2, participants received SRT from the researchers involved in the study. |
| 8. | Interviewer characteristics | Participants were informed about the study with transparency |
| **Domain 2: study design** | |  |
| Theoretical framework | |  |
| 9. | Methodological orientation and theory | The study used a two-phase mixed-methods feasibility design. Phase 1 used co-production methods. Phase 2 used a pilot case-series design. |
| Participant selection | | |
| 10. | Sampling | Phase 1 used convenience sampling of young people aged 12–18 with sleep and mood difficulties, including Lambeth YPAG input, plus clinician feedback. Phase 2 participants were recruited from Lambeth CAMHS |
| 11. | Method of approach | Young people were approached through CAMHS/co-production routes and routine clinical/research contacts. Phase 1 included an online questionnaire, YPAG presentation and one semi-structured young person interview. Phase 2 participants were approached through Lambeth CAMHS clinical pathways. |
| 12. | Sample size | Phase 1 included eleven young people and six clinicians. Phase 2 included six participants. |
| 13. | Non-participation | One of the participants refused as they preferred exclusive pharmacological management |
| Setting |  |  |
| 14. | Setting of data collection | Phase 1 data was collected remotely. Phase 2 data was collected face-to-face |
| 15. | Presence of non-participants | For the Phase 1 YPAG co-production session, facilitators for the group were present |
| 16. | Description of sample | Described in the results. |
| Data collection | | |
| 17. | Interview guide | Phase 1 feedback was structured around intervention adaptation priorities: understanding of SRT and perceived links between routines, sleep and mood; daily routine contexts and rhythm disruptors; preferences for intervention structure, delivery and monitoring; and barriers, facilitators and suggested adaptations. Phase 2 feedback focused on satisfaction, perceived usefulness, feasibility, and acceptability. |
| 18. | Repeat interviews | No repeat qualitative interviews were conducted. |
| 19. | Audio/visual recording | For phase 1, the YPAG even was transcribed. No recording for phase 2. |
| 20. | Field notes | “Field notes were used to capture contextual observations and reflections for internal use. |
| 21. | Duration | Sessions were 60 minutes long. |
| 22. | Data saturation | Given the small feasibility/co-production design, formal saturation was not the aim. |
| 23. | Transcripts returned | Transcripts were not returned or requested. |
| **Domain 3: analysis and findings** | | |
| Data analysis | | |
| 24. | Number of data coders | Qualitative feedback was coded by the three first authors. |
| 25. | Description of the coding tree | Phase 1 qualitative data were organised descriptively around core adaptation areas and main themes. Phase 2 feedback was organised around feasibility and acceptability |
| 26. | Derivation of themes | Themes were developed deductively from the intervention adaptation aims and feasibility objectives. |
| 27. | Software | NVivo 15 was used for qualitative data management/organisation. |
| 28. | Participant checking | Formal participant checking was not undertaken |
| Reporting |  |  |
| 29. | Quotations presented | Use anonymised participant quotations were presented to illustrate key themes. Consent was obtained for this. |
| 30. | Data and findings consistent | Findings were presented sequentially: first Phase 1 co-production findings and how these informed SRT adaptation; second Phase 2 feasibility, acceptability and exploratory clinical trajectories. |
| 31. | Clarity of major themes | Major themes were reported and how the results were structured explain. |
| 32. | Clarity of minor themes | Minor/ individual themes are included, and used for recommendations. |

**Supplementary eFigure 1. Patient and Public Involvement/co-production recruitment flyer used in Phase 1**

**Supplementary eFigure 2. Young person feedback form used during Phase 1 co-production**

**Supplementary eFigure 3. Recruitment flyer for the Social Rhythm Therapy pilot case series**

**Flyer for SRT’s pilot trial recruitment**

HAVE YOU BEEN DIAGNOSED WITH DEPRESSION?

**Supplementary eFigure 4. Weekly Social Rhythm Metric (SRM) form used for daily routine and mood monitoring in the Phase 2 STAR-D pilot**
